# Changes Induced by Heat Moisture Treatment in Wheat Flour and Pasta Rheological, Physical and Starch Digestibility Properties

**DOI:** 10.3390/gels9060449

**Published:** 2023-05-30

**Authors:** Mădălina Ungureanu-Iuga, Silvia Mironeasa

**Affiliations:** 1 Integrated Center for Research, Development and Innovation in Advanced Materials, Nanotechnologies, and Distributed Systems for Fabrication and Control (MANSiD), “Ştefan cel Mare” University of Suceava, 13th University Street, 720229 Suceava, Romania; madalina.iuga@usm.ro; 2 Mountain Economy Center (CE-MONT), “Costin C. Kiriţescu” National Institute of Economic Researches (INCE), Romanian Academy, 49th Petreni Street, 725700 Vatra Dornei, Romania; 3 Faculty of Food Engineering, “Ştefan cel Mare” University of Suceava, 13th University Street, 720229 Suceava, Romania

**Keywords:** hydrothermal treatment, common wheat pasta, texture, dough rheology, physical properties, resistant starch

## Abstract

Wheat is one of the main crops that is cultivated and consumed in the world. Since durum wheat is less abundant and more expensive than other types, pasta producers can use common wheat by applying various techniques to achieve the desired quality. A heat moisture treatment was applied to common wheat flour, and the effects on dough rheology and texture, and pasta cooking quality, color, texture, and resistant starch content were evaluated. The results revealed that heat moisture treatment temperature and moisture content induced a proportional increase in visco-elastic moduli, dough firmness, pasta cooking solids loss, and luminosity, as they were higher compared to the control. The breaking force of uncooked pasta decreased when the flour moisture content increased, while the opposite trend was observed for resistant starch content. The highest resistant starch values were obtained for the samples treated at the lowest temperature (60 °C). Significant correlations (*p* < 0.05) were obtained between some of the textural and physical characteristics analyzed. The studied samples can be grouped in three clusters characterized by different properties. Heat moisture treatment is a convenient physical modification of starch and flours that can be employed in the pasta industry. These results underline the opportunity to enhance common pasta processing and final product functionality by using a green and non-toxic technique to develop new functional products.

## 1. Introduction

Wheat is one of the main crops that is cultivated and consumed worldwide. There are many food products based of wheat flour, including bread, pasta, and crackers. Pasta is usually made of durum wheat semolina, but in some regions where the cultivation of this variety is small and the price is high, common wheat can be used for the manufacturing of pasta. The production of common wheat and spelt in Europe in 2021 was 130,807.96 thousand tonnes, while durum wheat accounted for only 7969.78 thousand tonnes [1]. Thus, one of the challenges faced by the processing industry and the researchers is to find solutions with regard to the manufacturing of pasta with superior nutritional value by using common wheat flour. Furthermore, the interest of consumers in functional products has increased of late, so the enhancement of existing types of foods is necessary.

A heat moisture treatment (HMT) is a non-polluting, cheap and non-toxic physical technique for starch and flour modification, and involves the heat-treatment of the material with a low moisture content (10–30%), and the temperature between the glass transition and gelatinization (90–120 °C) for a given time (15 min to 16 h) [2,3]. One of the advantages of HMT is related to the increase of resistant starch content, resulting in food products with a relatively slow digestion rate and a lower glycemic index [4]. Thus, the consumption of products with a high resistant starch content can contribute to the prevention of some diseases such as obesity, diabetes, and cardiovascular problems [4]. Starch is considered to be one of the main sources of carbohydrates in the human diet. This biopolymer presents a granular form, and it is widely found in cereals, legumes, tubers, and oilseeds, but native starch may have poor tolerance to technological processing [2]. The properties of starch can be ameliorated by using various techniques such as physical, chemical or enzymatic modifications, the most advantageous from the environmental and cost perspectives being the physical one [5].

As stated in the literature, HMT was shown to create disruptions on the granular, crystalline, lamellae, and helical structure, as well as to cause the molecular reorganization of starch from many sources, leading to changes in gel rheological behavior, functional properties, and final product quality [4,6,7,8].

Abhilasha et al. [8] demonstrated that HMT applied to wheat grains resulted in lower starch hydrolysis after oral-gastro-small intestinal digestion in vitro, with the treatment temperature determining the enhanced starch gelatinization properties. The HMT of A and B-type wheat starches caused modifications in the starch molecules, which induced the diversification of the crystal structure and changes in the physicochemical properties, a lower gel viscosity, higher solubility, and a slowly digestible starch content compared to the native samples, according to the data reported by Zhang et al. [7]. Bao et al. [4] revealed that the resistant starch content of rice starch treated by HMT was significantly higher compared to untreated samples; the gelatinization temperature increased, while an opposite effect was observed for the paste viscosity. Mann et al. [9] demonstrated a reduction of protein solubility and a less compact wheat dough structure, which was caused by the formation of gluten aggregates and by the linkages formed between the starch and proteins during HMT, these changes being more evident as the moisture content was higher due to the increased mobility of the molecules. Another study conducted by Liao et al. [10] revealed an increased dynamic viscoelastic moduli of pasta dough for vermicelli made of HMT potato starch, the increase being proportional to the moisture content growth, with pasta texture parameters such as hardness, springiness and chewiness increasing as well, as the amount of water was higher. The treatment of wheat flour with superheated steam resulted in the distortion of starch granules, the generation of aggregates between starch, gluten proteins and/or lipids, and the increase in the slowly digestible starch content to more than 14%, depending on the temperature applied, as reported by Ma et al. [11].

Considering all the facts listed above, the present study aimed to investigate the influence of HMT with different temperatures, times and moisture content of common wheat flour on the physical, textural and rheological properties of dough and pasta. To the best of our knowledge, there are few papers concerning the application of HMT on wheat flour and the subsequent use in pasta products. By examining the physical, textural, rheological properties and starch digestibility of wheat pasta dough and/or the final product, we aimed to underline the possibility to use this technique in common wheat pasta production in order to obtain final products of acceptable quality and with benefits for human health.

## 2. Results and Discussion

### 2.1. Rheological Dynamic Moduli and Dough Texture

**Figure 1:**
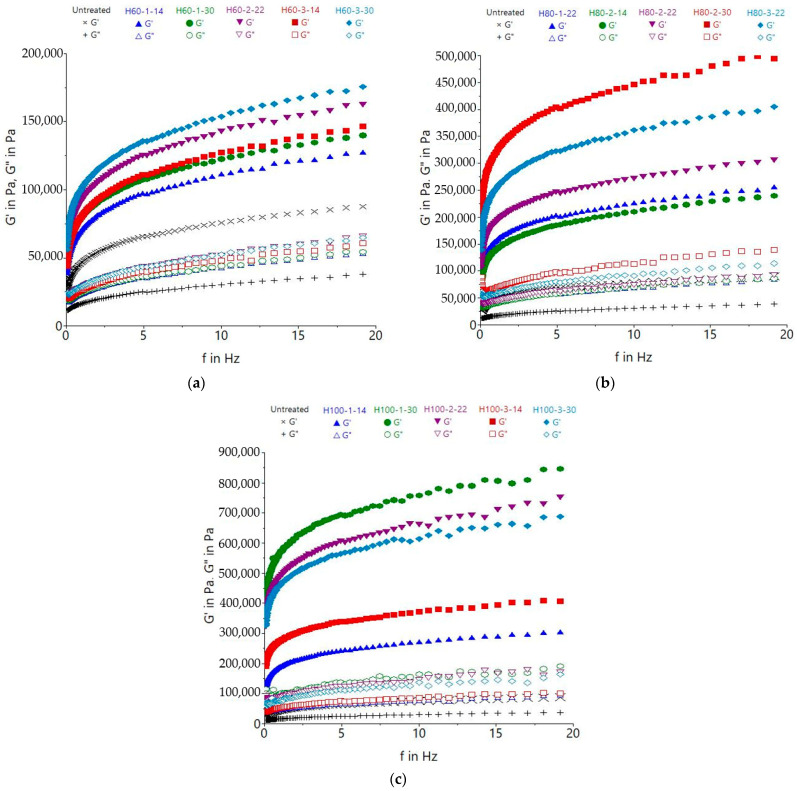
Dynamic moduli variation in the function of frequency for pasta dough made of treated wheat flour at (**a**) 60 °C, (**b**) 80 °C, and (**c**) 100 °C compared to untreated flour dough; the sample codification includes treatment (H for treated samples), followed by temperature (60, 80 or 100 °C), then the time (1, 2 or 3 h) and flour moisture (14, 22 or 30%). The uncooked sample served as the control, and therefore was not treated.

HMT generates changes in the starch and protein structure of wheat flour, resulting in a modification of the gel structure and the dough’s rheological behavior (Figure 1). All of the studied samples exhibited G′ values higher than G″, which means that they had a solid-like behavior, being more elastic than viscous.

The increase in treatment temperature caused a proportional increase of the elastic (G′) and viscous (G″) moduli values. All of the treated samples exhibited higher G′ and G″ compared to the untreated ones; generally, the gradual increase of moisture content was related to the higher visco-elastic moduli. A possible explanation of these variations could be the aggregation of gluten proteins as a result of structural deformations and –S–S– bond formation that occurs during HMT, leading to a polymer size increase and its fixation in a quite compact state, which will obstruct the unfolding and re-bonding upon the addition of water and the mixing [9]. The reinforcement effect of dough could also be related to the modification of the starch structure and hydrophobicity, which affects the interactions with gluten [9]. Villanueva et al. [12] reported an increase of elastic and viscous moduli when microwave treated rice flour was used in gluten-free bread, explaining that the rearrangements of the amylose and amylopectin chains probably contributed to the dough strengthening. Furthermore, the increase in the visco-elastic moduli could have been promoted by the exposure of hydrophilic groups (–COOH, –NH_2_) generated by HMT which led to a higher water-binding capacity [13].

Dough texture is directly influenced by the linkages between gluten proteins and starch, lipids, and other components of the matrix. HMT may affect the gluten gel structure, resulting in changes of pasta dough behavior during compression. Generally, dough firmness and springiness increased after HMT compared to the untreated sample, while the adhesiveness and resilience decreased, with some exceptions (Table 1). A higher treatment temperature led to a firmer dough with less adhesive and a lower resilience.

The increase of moisture content resulted in increased dough firmness when the time was at the minimum (1 h), and higher when the time was at its maximum (3 h), with lower resilience and adhesiveness (except for the samples treated for 3 h) being obtained. The springiness was affected in an irregular way by the time and moisture. The behavior of the wheat-based dough is dictated by the starch-gluten interactions and the gel formed by them. Starch grains act as filling agents and are usually encompassed into the gluten gel. An increased filling degree can be obtained when the starch grains are smaller or have irregular forms, leading to a uniform interplay between the two main components—starch and gluten in dough, and could therefore enhance its stability [14]. The influence of HMT may emerge from the special distribution of treated starch granules in the gluten reticular network that affects gel packing strength [15]. –S–S– linkages are essential for maintaining the gluten gel tertiary structure and are important for the development of dough’s visco-elastic properties. Starch crystallinity modifies after HMT, and its exposed hydroxyls may be linked to the amino groups of gluten through hydrogen bonding, improving thus the strength of the gel [15].

### 2.2. The Physico-Chemical Properties and Texture of Pasta

**Figure 2:**
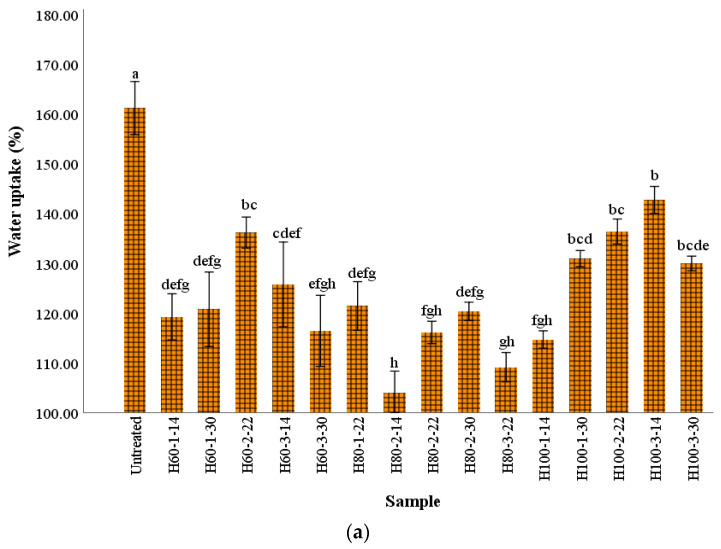

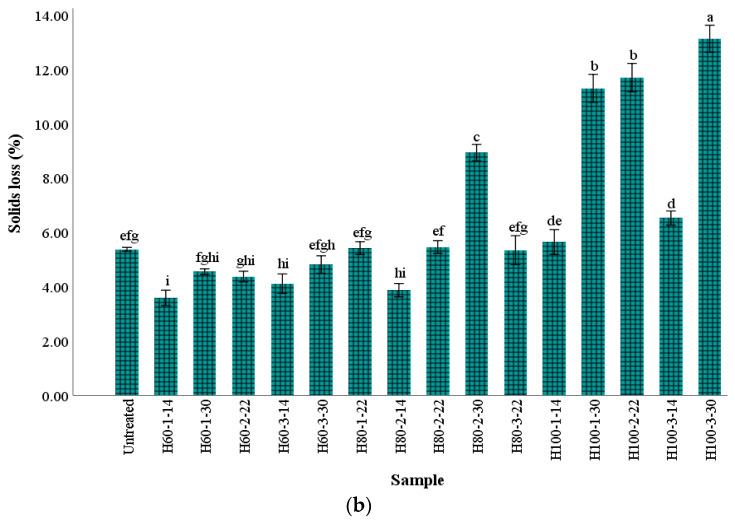
Pasta water uptake (**a**) and solids loss (**b**) during cooking; bars followed by different letters are significantly different at *p* < 0.05; sample codification includes treatment (H for treated samples), followed by temperature (60, 80 or 100 °C), and then the time (1, 2 or 3 h) and flour moisture (14, 22 or 30%), with the uncooked sample being the control, and therefore without any treatment; ^a–i^ columns with different letters are significantly different at *p* < 0.05.

The modifications of starch and proteins that occurred during the wheat flour HMT influenced pasta water uptake and solids loss (Figure 2). The water uptake of the untreated sample was significantly higher (*p* < 0.05) compared to pasta made of HMT flour. The HMT moisture content increase resulted in the higher water absorption of pasta, except for the samples treated for 3 h (Figure 2a). Li et al. [16] also reported the higher water uptake of buckwheat noodles and explained this behavior as resulting from the gelatinization process that occurs during HMT. In addition, the interactions among the water and carbohydrate polar groups, along with other polar molecules in flour, could also have been involved in the increase in water uptake.

The lowest values for water uptake were obtained for pasta made of flour treated at 80 °C. Chung et al. [17] also reported a higher cooking loss and water absorption of wheat noodles with brown rice heat moisture treated at greater moisture levels, suggesting the alteration of the starch structure, which made the dough more susceptible to water hydration. By increasing the severity of the treatment (with a temperature higher than 80 °C and a moisture greater than 22%), the solid loss during pasta cooking exhibited greater values compared to the untreated sample. In all cases, proportional increases of solid loss with moisture content and treatment temperature increases were obtained, while the time seemed to have a negligible impact on this parameter (Figure 2b). The solids loss of pasta changes could be related to the partial gelatinization of starch, and to the higher dissolution of soluble compounds including the amylose promoted by the flour HMT [18]. Furthermore, protein denaturation could be responsible for the release of solids during cooking by modifying the gluten gel structure [19].

The chromatic parameters of uncooked pasta are listed in Table 2. The luminosity (L*) and hue angle (H*) generally increased when the temperature was higher, with the values being greater than of the untreated samples only for an HMT of more than 80 °C. A higher moisture content resulted in more luminous pasta, with all of the samples exhibiting a yellow-green nuance (H* values positioned in the second quadrant).

The green nuance of the pasta decreased as the temperature and time were higher, while an opposite trend occurred when the moisture increased. The yellow nuance was less pronounced in samples made of flour treated at higher temperatures and for a longer time, with the moisture content also generating a decreasing trend (Table 2). Vadlamani et al. [20] obtained brighter wheat noodles when HMT was applied, which was probably the result of polyphenol oxidase activity modifications, which might be similar to our findings. According to Wang et al. [6], the decrease in the green nuance of pasta with HMT conditions and the reduction of yellowness could be related to the non-enzymatic reactions that may occur during HMT.

The texture of pasta provides important quality information, and is directly influenced by the structure of the components of the dough. The breaking force of uncooked pasta had lower values when HMT was applied at greater moisture levels (Table 3), while temperature and time affected this parameter in an irregular way.

The elasticity of pasta was not significantly (*p* > 0.05) affected by HMT, while gumminess and firmness decreased as the temperature and moisture content increased (except for the samples treated for 3 h), with a similar trend being obtained as the time increased. Huang et al. [18] also reported the lower hardness and elasticity of fresh noodles made of superheated steam wheat flour compared to the control. After HMT, protein denaturation may happen, causing the insufficient development of the gluten gel matrix, which becomes unable to provide enough physical strength for the pasta [18]. Kaur et al. [21] demonstrated that the textural parameters of noodles are dependent not only on the properties of the flour proteins, but also on the characteristics of the starches. Bruneel et al. [22] revealed that the disulfide linked protein gel matrix found in pasta is responsible for the particles’ shear resistance, starch swelling limitations, and subsequent leaching. The protein polymerisation during the processing is essential for obtaining high quality pasta, because when the proteins are polymerised too strongly, they have insufficient capacity to manage the starch swelling during cooking [22], resulting in a stickier and less firm pasta.

**Figure 3:**
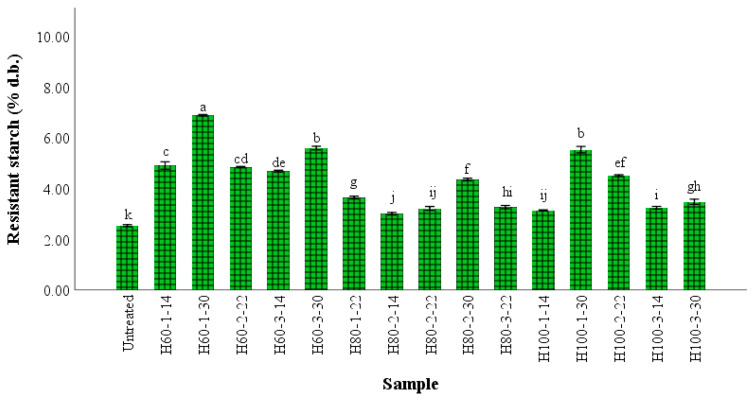
The resistant starch content of pasta samples. Bars followed by different letters are significantly different at *p* < 0.05; the sample codification includes treatment (H for treated samples), followed by temperature (60, 80 or 100 °C), and then time (1, 2 or 3 h) and flour moisture (14, 22 or 30%), with the uncooked sample being the control, and therefore not receiving any treatment; ^a–k^ columns with different letters are significantly different at *p* < 0.05.

One of the main changes in wheat flour functionality after HMT is related to the slowing of the digestibility of starch. The increase of resistant starch in treated wheat pasta compared to untreated pasta is shown in Figure 3. Protein denaturation occurs when HMT is applied to wheat flour, with the modified molecules being able to adhere on the starch grain aggregates surface to create barriers that slow down the digestive activity of the enzymes [13]. Furthermore, the formation of amylose-lipid complexes could be another explanation for the RS content increase, since amylose has a helical shape that can encompass fat or other hydrophobic molecules of a small size [13].

The moisture content of the increased flour resulted in significantly (*p* < 0.05) greater amounts of resistant starch in the final product, with the best results being observed at the lowest temperature (60 °C). The effects of changes of the starch grains microstructure on its hydrolysis have been investigated in previous studies, and it was demonstrated that the intact microstructure of whole grains limits the enzymatic action of starch [8]. According to Bao et al. [4], there are many factors impacting the influence of HMT on resistant starch content: the time and temperature of the treatment, the botanical origin of the starch, the moisture content of the sample, the interactions between amylose–lipid, amylose–amylose chains, amylose–amylopectin chains, and amylopectin–amylopectin chains. Hung et al. [23] reported a higher level of resistant starch in wheat cookies when HMT potato starch was added compared to the addition of native potato starch. The process of starch digestibility changes that occur during HMT could be the result of modifications in starch multi-scale structures, since the increase of the number of short-range ordered structures, helical structures, and B- or V type crystals could result in a significant lowering of the digestibility of starch [24].

### 2.3. Relationships between Variables

**Figure 4:**
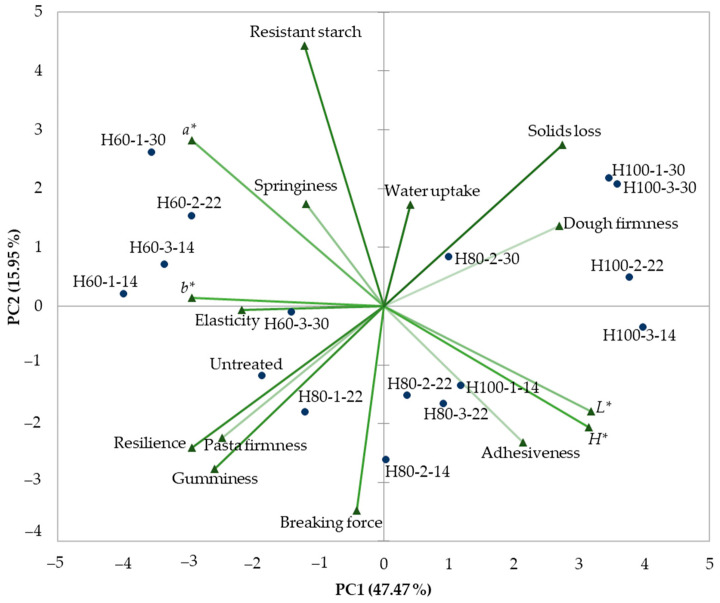
Principal component analysis (PCA) variables and biplot scores. The sample codification includes treatment (H for treated samples), followed by temperature (60, 80 or 100 °C), and then the time (1, 2 or 3 h) and flour moisture (14, 22 or 30%), with uncooked samples being the control and therefore not receiving any treatment.

The relationships between the studied variables were evaluated by means of Pearson correlation coefficients, and Principal Component Analysis (PCA). The first component (PC1) accounted for 47.47% of the data variation, while the second component accounted for 15.95% of the variability (Figure 4). PC1 was associated with the color parameters, dough firmness, and pasta elasticity, while the resistant starch content and breaking force were related to PC2. Taking into account their position close to the center, springiness, water uptake, and elasticity contribute less to the data variability than the other variables do.

The significant correlations (*p* < 0.05) obtained between the textural, physical and chemical characteristics are detailed below (Table 4). The dough firmness was negatively correlated with all cooked pasta textural parameters, while with solids loss a positive relation was obtained (*r* = 0.65). Evlice [25] also obtained a significant correlation between durum wheat pasta firmness and cooking loss.

The resistant starch content was negatively correlated with dough adhesiveness (Table 4), which agreed with the findings of Kolarič et al. [26] that adhesiveness is related to the amount of starch and starch gelatinization, while with solids loss a positive relationship was obtained. Positive correlations were observed between dough resilience and the pasta breaking force, gumminess and firmness, and a negative correlation was found with solid loss (*r* = −0.84). Strong correlations were observed between all of the color parameters, while solid loss was positively correlated with pasta elasticity and negatively correlated with gumminess and firmness. Significant but weak correlations (*p* < 0.05) were obtained for the breaking force with resistant starch content and solids loss (Table 4). Similar to our results, Biernacka at al. [27] obtained significant correlations between color parameters (L*, a* and b*) and solids loss during pasta cooking.

### 2.4. Cluster Analysis

**Figure 5:**
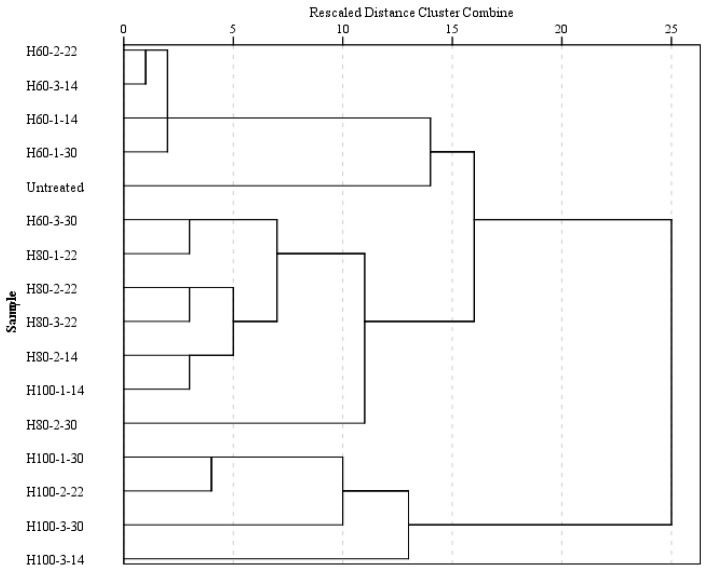
Hierarchical cluster analysis (HCA) dendrogram: the sample codification includes treatment (H for treated samples), followed by temperature (60, 80 or 100 °C) and then the time (1, 2 or 3 h) and flour moisture (14, 22 or 30%), with the uncooked sample being the control and therefore not receiving any treatment.

A hierarchical cluster analysis revealed that the studied samples can be grouped in three clusters (Figure 5): the first one comprised the samples treated at 60 °C (except the one with 30% moisture treated for 3 h), and the untreated one, the second cluster was formed by the samples treated at 80 °C, in addition to the sample with the most severe treatment at 60 °C and the one treated at 100 °C for 1 h at 14% moisture; the last cluster is represented by the remaining samples treated at 100 °C.

The characteristics of the three groups of samples are listed in Table 5. Significant differences (*p* < 0.05) between the groups were observed in terms of dough firmness, adhesiveness, resilience, pasta L*, a*, b*, H*, gumminess, firmness, water uptake, and solids loss.

The first group was characterized by the highest dough resilience, green and yellow nuances, pasta gumminess and firmness, and a resistant starch content, while the lowest values were observed for dough firmness, adhesiveness, luminosity, and solids loss. The second group presented the highest breaking force and the lowest resistant starch content, while the last group was characterized by the highest dough firmness, adhesiveness, luminosity, hue angle, water uptake, solids loss, and the lowest dough resilience, springiness, green and yellow nuances, pasta gumminess, and firmness.

## 3. Conclusions

Heat moisture treatment can be a useful tool to modify the technological and functional properties of wheat flour in order to obtain superior pasta products with an enhanced resistant starch content. The changes produced by HMT in wheat protein and starch gel structure determined the variations of the rheological and textural properties of dough, depending on the temperature, moisture and time used for the flour treatment. These changes are directly related to the quality of the final product, with the HMT conditions being responsible for color, texture and resistant starch content modifications whose magnitude depends on the treatment severity. The obtained results suggested that a moderate HMT temperature (60–80 °C), moisture content (about 22%), and time (1–2 h) can be recommended to obtain pasta with the highest resistant starch content and with the minimum negative effects to the final product quality. These results can be helpful for industry and could be a starting point for further research concerning the development of functional wheat products. More investigations concerning the molecular interactions that occur during HMT and the effects on the sensory characteristics of the final product are necessary.

## 4. Materials and Methods

### 4.1. Heat Moisture Treatment and Pasta Production

**Figure 6:**
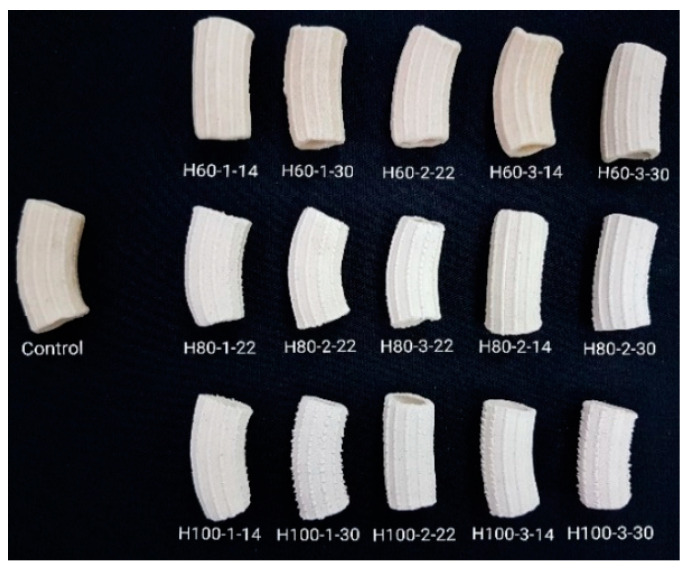
Pasta samples from untreated (control) and treated flour (HT-t-m), where H: hydrothermal treatment, T: temperature (60, 80, 100 °C), t: time (1, 2, 3 h), and m: moisture (14, 22, 30%) [29].

The heat moisture treatment (HMT) of common wheat (*Triticum aestivum*) 650 type flour was done according to the methodology described in our previous study [28]. Briefly, the moisture of wheat flour samples was adjusted to 14, 22 or 30% by incorporating small portions of water, followed by continuous stirring, and then the samples were left in sealed containers for 24 h at 22 °C to allow for moisture equalization. The conditioned flour was heated at 60, 80 or 100 °C for 1, 2 or 3 h in hermetical glass containers in a convection oven (Helpan Forni, model POWER SNACK 4M, Thiene, Italy), after which they were cooled to room temperature in open air, dried at 40 °C for 12 h in the convection oven, and grinded and sieved to achieve particle sizes < 300 μm (Figure 6). The codification (HT-t-m) of the hydrothermally treated samples (H) included the factors of temperature (marked with T), time (marked with t), and moisture (marked with m), and the sample of untreated wheat flour pasta is the control. The experimental matrix was obtained by using a central composite design [28].

**Figure 7:**
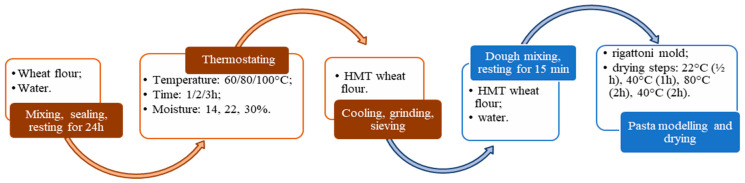
Samples preparation: wheat flour treatment (brown) and pasta production (blue).

Common wheat pasta made of untreated (control) and treated wheat flour were produced by mixing the flour in a Kitchen Aid device (Whirlpool Corporation, Benton Harbor, MI, USA) with an adequate quantity of water calculated to obtain a dough with 40% moisture. The dough was left for 15 min at 22 °C in sealed containers, after which the pasta was modeled by using a rigatoni mold of a Kitchen Aid machine. The methodology of Bergman et al. [30] was employed for the drying of the pasta (Figure 7). The samples were left for ½ h in open air at 22 °C, after which they were put in a convection oven for 1 h at 40 °C, and the temperature was raised for 2 h to 80 °C and in the last step the temperature was returned for to 40 °C for 2 h.

### 4.2. Rheological Dynamic Moduli Analysis

The viscous (G′) and elastic (G″) modulus of dough were measured after laminating the sample and leaving it for 30 min at 22 °C in closed containers to allow for internal strain removal. A frequency sweep test was employed and a Thermo-HAAKE, MARS 40 (Karlsruhe, Germany) dynamic rheometer with parallel plates of 40 mm diameter was used. The gap was 3 mm and the sample external edge was covered with Vaseline to avoid moisture evaporation. The frequency varied between 0.1 and 20 Hz, the strain was 15 Pa (situated in the linear viscoelastic region (LVR) earlier tested), and the working temperature was 20 °C.

### 4.3. Dough Texture Parameter Determination

A texture analysis was performed, as previously described in our study [31]. Dough firmness, adhesiveness, resilience and springiness were determined by double compression on a Perten TVT-6700 texturometer (Perten Instruments, Hägersten, Sweden). For this purpose, dough balls weighting 50 g were compressed at 50% of their height, with a velocity of 5.0 mm/s and a trigger force of 20 g, with a cylindrical probe of 3.5 cm diameter.

### 4.4. CIELab Chromatic Parameter Determination

The chromatic parameters such as luminosity (L*), red or green color (a*), and the yellow or blue color (b*) of uncooked pasta were determined by reflectance. The hue angle (H*) was calculated as the arctangent from the ration b*/a*. The CIE Lab system and a Konica Minolta CR-400 (Tokyo, Japan) device were used.

### 4.5. Pasta Water Uptake and Solids Loss Evaluation

The pasta samples’ solids loss and water uptake during cooking were evaluated by the gravimetric method, in line with that of Gimenez et al. [32]. Briefly, 10 g of sample was boiled in 200 mL of water for the optimum cooking time previously determined, and the weight of the pasta and solids released were recorded.

### 4.6. Pasta Textural Parameter Achievement

The texture of the pasta was analyzed as previously described [33]. The breaking force of uncooked pasta was achieved by means of a Perten TVT-6700 device (Perten Instruments, Stockholm, Sweden), supplied with an aluminum break rig set, the velocity of the test being of 3 mm/s, with a trigger force of 50 g [33].

The elasticity, gumminess and firmness of the boiled pasta were achieved on the same device by applying a double compression with a cylindric probe of 35 mm diameter. The compression was set at 50% of the sample height, the test velocity was 5.0 mm/s, and the trigger force was 20 g [33].

### 4.7. Resistant Starch Content Determination

The amount of resistant starch found in 100 g of cooked pasta (reported to dry basis) was quantified by applying the international AOAC 2017.16 standard method, which involved the use of a Megazyme kit [31]. Briefly, the sample was digested with α-amylase and amyloglucosidase for 2 h, after which it was submitted to a second digestion with only amyloglucosidase, and the glucose released was determined spectrophotometrically at 510 nm by using a glucose oxidase/peroxidase (GOPOD) reagent [31].

### 4.8. Statistical Processing of the Experimental Data

All of the analyses were performed at least in triplicate for each sample. The influence of HMT on the dough and pasta properties were determined by the use of a one-way ANOVA and a Tukey’s test (*p* < 0.05). A Principal Component Analysis (PCA) with Pearson correlations was employed to determine the similarities and dissimilarities between variables.

A hierarchical cluster analysis (HCA) using average linkage between groups was performed to determine how the pasta samples can be grouped and characterized according to their considered properties. The data were z-transformed in order to avoid the effects of significant differences in mass concentration values. The groups of pasta samples were formed by using the squared geometric Euclidian distance. The results were presented as a dendrogram comprising a hierarchical structure. The differences between clusters were evaluated by applying solution an analysis of variance (ANOVA) to the cluster solution.

The statistical processing of data was performed with the use of XLSTAT for Excel 2021 (Addinsoft, New York, NY, USA) software and SPSS (IBM, New York, NY, USA) trial version software.

## Figures and Tables

**Table 1:** The textural properties of pasta dough.

Sample	Dough Firmness (N)	Adhesiveness (J)	Resilience (adim.)	Springiness (adim.)
Untreated	19.08 ± 0.17 ^hi^	−14.51 ± 2.33 ^bc^	1.20 ± 0.00 ^abcd^	0.9962 ± 0.0009 ^bcd^
H60-1-14	17.02 ± 0.56 ^i^	−32.30 ± 2.32 ^g^	1.31 ± 0.07 ^a^	0.9970 ± 0.0004 ^abcd^
H60-1-30	21.19 ± 1.01 ^gh^	−42.19 ± 1.67 ^h^	1.16 ± 0.02 ^bcd^	0.9974 ± 0.0005 ^abc^
H60-2-22	22.50 ± 0.36 ^g^	−44.74 ± 4.69 ^hi^	1.26 ± 0.05 ^ab^	0.9977 ± 0.0005 ^abc^
H60-3-14	20.77 ± 0.68 ^gh^	−49.52 ± 3.37 ^i^	1.26 ± 0.02 ^ab^	0.9974 ± 0.0005 ^abc^
H60-3-30	16.72 ± 1.00 ^i^	−15.70 ± 3.19 ^cd^	1.18 ± 0.05 ^bcd^	0.9970 ± 0.0010 ^abcd^
H80-1-22	21.19 ± 0.17 ^gh^	−8.33 ± 0.76 ^ab^	1.12 ± 0.04 ^cd^	0.9977 ± 0.0008 ^abc^
H80-2-14	23.76 ± 0.83 ^g^	−15.06 ± 2.67 ^bc^	1.21 ± 0.04 ^abc^	0.9950 ± 0.0003 ^d^
H80-2-22	33.59 ± 0.85 ^e^	−23.47 ± 0.93 ^ef^	1.14 ± 0.02 ^cd^	0.9981 ± 0.0003 ^ab^
H80-2-30	55.28 ± 1.05 ^b^	−22.75 ± 2.17 ^de^	1.14 ± 0.01 ^bcd^	0.9984 ± 0.0003 ^a^
H80-3-22	31.23 ± 1.54 ^e^	−30.27 ± 3.80 ^fg^	1.12 ± 0.01 ^cd^	0.09964 ± 0.0010 ^abcd^
H100-1-14	33.60 ± 1.90 ^e^	−13.45 ± 1.21 ^bc^	1.09 ± 0.04 ^d^	0.9961 ± 0.0008 ^bcd^
H100-1-30	64.09 ± 1.52 ^a^	−23.45 ± 0.60 ^ef^	0.87 ± 0.01 ^e^	0.9958 ± 0.0014 ^cd^
H100-2-22	47.22 ± 1.34 ^d^	−13.26 ± 0.29 ^bc^	0.87 ± 0.02 ^e^	0.9967 ± 0.0000 ^abcd^
H100-3-14	51.61 ± 0.52 ^c^	−19.20 ± 1.69 ^cde^	0.94 ± 0.04 ^e^	0.9962 ± 0.0008 ^abcd^
H100-3-30	27.50 ± 1.49 ^f^	−4.95 ± 1.25 ^a^	0.20 ± 0.07 ^f^	0.9969 ± 0.0008 ^abcd^

^a–i^ mean values followed by different letters in the same column are significantly different at *p* < 0.05; sample codification includes treatment (H for treated samples), followed by temperature (60, 80 or 100 °C), followed by the time (1, 2 or 3 h) and flour moisture (14, 22 or 30%), with the uncooked sample being the control and therefore not receiving any treatment.

**Table 2:** Chromatic parameters of uncooked pasta.

Sample	L* (adim.)	a* (adim.)	b* (adim.)	H* (°)
Untreated	75.57 ± 0.62 ^f^	−2.09 ± 0.13 ^d^	21.81 ± 0.08 ^d^	178.52 ± 0.01 ^g^
H60-1-14	71.07 ± 0.38 ^i^	−1.38 ± 0.08 ^b^	24.47 ± 0.06 ^b^	178.49 ± 0.00 ^i^
H60-1-30	72.32 ± 0.14 ^h^	−0.86 ± 0.04 ^a^	23.09 ± 0.05 ^c^	178.47 ± 0.00 ^j^
H60-2-22	71.69 ± 0.31 ^hi^	−1.62 ± 0.09 ^c^	22.87 ± 0.05 ^c^	178.50 ± 0.00 ^h^
H60-3-14	73.93 ± 0,58 ^g^	−1.57 ± 0,12 ^bc^	25.44 ± 0.40 ^a^	178.49 ± 0.00 ^hi^
H60-3-30	76.50 ± 0.12 ^ef^	−2.36 ± 0.03 ^e^	19.91 ± 0.01 ^ef^	178.55 ± 0.00 ^f^
H80-1-22	77.89 ± 0.38 ^d^	−2.66 ± 0.09 ^f^	21.74 ± 0.77 ^d^	178.55 ± 0.01 ^f^
H80-2-14	80.59 ± 0.37 ^c^	−3.32 ± 0.03 ^gh^	20.63 ± 0.05 ^e^	178.59 ± 0.00 ^d^
H80-2-22	81.88 ± 0.32 ^b^	−3.48 ± 0.01 ^h^	18.73 ± 0.57 ^gh^	178.61 ± 0.00 ^c^
H80-2-30	76.76 ± 0.26 ^e^	−2.42 ± 0.07 ^e^	16.65 ± 0.03 ^j^	178.57 ± 0.00 ^e^
H80-3-22	80.29 ± 0.23 ^c^	−3.32 ± 0.10 ^gh^	18.37 ± 0.36 ^hi^	178.61 ± 0.00 ^c^
H100-1-14	80.61 ± 0.18 ^c^	−3.94 ± 0.05 ^i^	19.54 ± 0.07 ^fg^	178.63 ± 0.00 ^b^
H100-1-30	82.49 ± 0.12 ^b^	−3.18 ± 0.07 ^g^	16.68 ± 0.08 ^j^	178.62 ± 0.00 ^c^
H100-2-22	84.59 ± 0.25 ^a^	−3.91 ± 0.03 ^i^	17.80 ± 0.05 ^i^	178.65 ± 0.00 ^a^
H100-3-14	84.27 ± 0.14 ^a^	−4.05 ± 0.04 ^i^	19.83 ± 0.11 ^ef^	178.63 ± 0.00 ^b^
H100-3-30	80.21 ± 0.21 ^c^	−2.80 ± 0.04 ^f^	18.51 ± 0.17 ^hi^	178.58 ± 0.00 ^de^

^a–j^ mean values followed by different letters in the same column are significantly different at *p* < 0.05; the sample codification includes treatment (H for treated samples), followed by temperature (60, 80 or 100 °C), and then the time (1, 2 or 3 h) and flour moisture (14, 22 or 30%), with the uncooked sample being the control, and therefore not receiving any treatment.

**Table 3:** Textural parameters of pasta.

Sample	Breaking Force (N)	Elasticity (%)	Gumminess (N)	Pasta Firmness (N)
Untreated	41.03 ± 1.75 ^efgh^	99.87 ± 0.02 ^ab^	48.76 ± 2.18 ^a^	74.05 ± 0.91 ^a^
H60-1-14	41.28 ± 3.04 ^efgh^	99.87 ± 0.01 ^ab^	46.79 ± 0.23 ^a^	65.86 ± 0.92 ^b^
H60-1-30	38.55 ± 1.08 ^ghi^	99.88 ± 0.01 ^a^	34.47 ± 1.33 ^bc^	53.62 ± 0.73 ^de^
H60-2-22	40.47 ± 0.66 ^fgh^	99.87 ± 0.00 ^a^	35.93 ± 1.75 ^bc^	48.61 ± 3.51 ^ef^
H60-3-14	46.29 ± 2.22 ^abcd^	99.86 ± 0.01 ^ab^	32.30 ± 0.56 ^c^	60.41 ± 3.18 ^bc^
H60-3-30	42.97 ± 2.36 ^cdef^	99.87 ± 0.00 ^a^	34.91 ± 1.49 ^bc^	60.64 ± 4.23 ^bc^
H80-1-22	50.74 ± 0.09 ^a^	99.86 ± 0.01 ^ab^	44.20 ± 0.32 ^a^	61.60 ± 1.56 ^bc^
H80-2-14	44.73 ± 0.31 ^bcdef^	99.87 ± 0.01 ^a^	37.59 ± 0.75 ^b^	52.83 ± 2.49 ^de^
H80-2-22	45.43 ± 1.63 ^bcde^	99.87 ± 0.01 ^a^	37.06 ± 0.25 ^b^	56.62 ± 0.30 ^cd^
H80-2-30	41.86 ± 1.25 ^defg^	99.81 ± 0.07 ^ab^	35.97 ± 0.19 ^bc^	47.24 ± 1.96 ^ef^
H80-3-22	48.05 ± 0.84 ^ab^	99.82 ± 0.07 ^ab^	35.75 ± 0.75 ^bc^	45.45 ± 0.86 ^f^
H100-1-14	35.21 ± 0.81 ^ij^	99.86 ± 0.01 ^ab^	36.92 ± 1.42 ^bc^	52.11 ± 0.98 ^de^
H100-1-30	33.88 ± 0.15 ^j^	99.85 ± 0.01 ^ab^	24.43 ± 4.30 ^d^	27.25 ± 1.93 ^g^
H100-2-22	44.42 ± 1.38 ^bcdef^	99.85 ± 0.01 ^ab^	22.89 ± 1.23 ^d^	25.28 ± 2.63 ^g^
H100-3-14	47.03 ± 1.89 ^abc^	99.77 ± 0.09 ^b^	16.04 ± 0.95 ^e^	21.54 ± 0.96 ^g^
H100-3-30	36.94 ± 0.90 ^hij^	99.84 ± 0.00 ^ab^	16.85 ± 1.31 ^e^	23.07 ± 1.58 ^g^

^a–j^ mean values followed by different letters in the same column are significantly different at *p* < 0.05; samples codification includes treatment (H for treated samples), followed by temperature (60, 80 or 100 °C), and then the time (1, 2 or 3 h) and flour moisture (14, 22 or 30%), with the uncooked sample being the control and therefore not receiving any treatment.

**Table 4:** Pearson correlation coefficients.

Variables	Dough Firmness	Adhesi-Veness	Resilience	Springiness	L*	a*	b*	H*	Breaking Force	Elasticity	Gumminess	Pasta Firmness	Resistant Starch	Water Uptake	Solids Loss
Dough firmness	1.00														
Adhesiveness	0.19	1.00													
Resilience	−0.33 *	−0.51 **	1.00												
Springiness	−0.09	−0.26	0.14	1.00											
L*	0.65 **	0.61 **	−0.50 **	−0.35 *	1.00										
a*	−0.57 **	−0.62 **	0.36 **	0.35 *	−0.95 **	1.00									
b*	−0.75 **	−0.56 **	0.50 **	0.12	−0.75 **	0.70 **	1.00								
H*	0.68 **	0.61 **	−0.42 **	−0.31 *	0.96 **	−0.98 **	−0.82 **	1.00							
Breaking force	−0.20	−0.01	0.31 *	0.15	0.10	−0.13	0.18	0.04	1.00						
Elasticity	−0.48 **	−0.12	0.23	0.13	−0.35 *	0.33 *	0.34 *	−0.35 *	−0.12	1.00					
Gumminess	−0.57 **	−0.15	0.72 **	0.10	−0.59 **	0.44 **	0.45 **	−0.49 **	0.16	0.41 **	1.00				
Pasta firmness	−0.71 **	−0.24	0.73 **	0.17	−0.66 **	0.54 **	0.60 **	−0.62 **	0.20	0.46 **	0.92 **	1.00			
Resistant starch	−0.01	−0.52 **	0.14	0.25	−0.46 **	0.59 **	0.21	−0.49 **	−0.31 *	0.20	−0.10	−0.02	1.00		
Water uptake	0.14	0.07	−0.19	−0.03	0.00	0.07	0.07	−0.07	−0.12	−0.12	−0.16	−0.13	−0.11	1.00	
Solids loss	0.65 **	0.46 **	−0.84 **	−0.05	0.56 **	−0.40 **	−0.70 **	0.53 **	−0.36 *	−0.24	−0.68 **	−0.76 **	0.00	0.27	1.00

* Significant at *p* < 0.05, ** significant at *p* < 0.01; samples codification includes treatment (H for treated samples), followed by temperature (60, 80 or 100 °C), and then the time (1, 2 or 3 h) and flour moisture (14, 22 or 30%), with the uncooked sample being the control and therefore not receiving any treatment.

**Table 5:** Characteristics of the sample groups.

Characteristic	Group	Mean	Standard Deviation	Minimum	Maximum	*F*-Value ^†^
Dough firmness (N)	1	20.11 ^b^	2.11	17.02	22.50	6.59 *
	2	30.77 ^ab^	12.62	16.72	55.28	
	3	47.60 ^a^	15.19	27.50	64.09	
Adhesiveness (J)	1	−36.65 ^b^	13.88	−49.52	−14.51	6.63 *
	2	−18.44 ^a^	7.42	−30.27	−8.33	
	3	−15.21 ^a^	8.02	−23.45	−4.95	
Resilience (adim.)	1	1.24 ^a^	0.06	1.16	1.31	11.02 **
	2	1.14 ^a^	0.04	1.09	1.21	
	3	0.72 ^b^	0.35	0.20	0.94	
Springiness (adim.)	1	0.9971 ^a^	0.0006	0.9962	0.9977	0.78
	2	0.9970 ^a^	0.0012	0.9950	0.9984	
	3	0.9964 ^a^	0.0005	0.9958	0.9969	
L* (adim.)	1	72.92 ^c^	1.83	71.07	75.57	28.96 **
	2	79.22 ^b^	2.13	76.50	81.88	
	3	82.89 ^a^	2.01	80.21	84.59	
a* (adim.)	1	−1.50 ^b^	0.44	−2.09	−0.86	17.19 **
	2	−3.07 ^a^	0.60	−3.94	−2.36	
	3	−3.49 ^a^	0.59	−4.05	−2.80	
b* (adim.)	1	23.53 ^a^	1.42	21.81	25.44	16.64 **
	2	19.37 ^b^	1.65	16.65	21.74	
	3	18.21 ^b^	1.32	16.68	19.83	
H* (°)	1	178.49 ^b^	0.02	178.47	178.52	25.98 **
	2	178.59 ^a^	0.03	178.55	178.63	
	3	178.62 ^a^	0.03	178.58	178.65	
Breaking force (N)	1	41.53 ^a^	2.87	38.55	46.29	0.85
	2	44.14 ^a^	4.95	35.21	50.74	
	3	40.57 ^a^	6.18	33.88	47.03	
Elasticity (%)	1	99.87 ^a^	0.01	99.86	99.88	2.94
	2	99.85 ^a^	0.03	99.81	99.87	
	3	99.83 ^a^	0.04	99.77	99.85	
Gumminess (N)	1	39.65 ^a^	7.56	32.30	48.76	19.55 **
	2	37.48 ^a^	3.10	34.91	44.20	
	3	20.05 ^b^	4.22	16.04	24.43	
Pasta firmness (N)	1	60.51 ^a^	10.01	48.61	74.05	32.51 **
	2	53.78 ^a^	6.22	45.45	61.60	
	3	24.28 ^b^	2.51	21.54	27.25	
Resistant starch (% d.b.)	1	4.77 ^a^	1.54	2.53	6.88	1.10
	2	3.74 ^a^	0.93	3.00	5.59	
	3	4.19 ^a^	1.05	3.24	5.53	
Water uptake (%)	1	132.60 ^a^	17.28	119.21	161.14	6.17 *
	2	114.56 ^b^	6.18	103.91	121.39	
	3	134.98 ^a^	5.87	129.95	142.70	
Solids loss (%)	1	4.39 ^b^	0.65	3.58	5.37	15.24 **
	2	5.63 ^b^	1.57	3.86	8.92	
	3	10.65 ^a^	2.86	6.52	13.11	

**^†^** ANOVA results for the comparison between groups (** significant at *p* < 0.01,* significant at *p* < 0.05); the mean values followed by different letters in the same column are significantly different at *p* < 0.05.

## Data Availability

The experimental data are available from the corresponding author upon reasonable request.

## References

[1] EUROSTAT Crop production in EU Standard Humidity. https://data.europa.eu/data/datasets/u33k8gi1mfygn7hyhunhg?locale=en.

[2] Schafranski K., Cristina V., Gustavo L. (2021). Impacts and potential applications: A review of the modification of starches by heat-moisture treatment (HMT). Food Hydrocoll..

[3] Zavareze R., Renato A., Dias G. (2011). Impact of heat-moisture treatment and annealing in starches: A review. Carbohydr. Polym..

[4] Bao J., Zhou X., Hu Y., Zhang Z. (2022). Resistant starch content and physicochemical properties of non-waxy rice starches modified by pullulanase, heat-moisture treatment, and citric acid. J. Cereal Sci..

[5] Tarahi M., Shahidi F., Hedayati S. (2022). Physicochemical, Pasting, and Thermal Properties of Native Corn Starch–Mung Bean Protein Isolate Composites. Gels.

[6] Wang Q., Li L., Liu C., Zheng X. (2022). Heat-moisture modified blue wheat starch: Physicochemical properties modulated by its multi-scale structure. Food Chem..

[7] Zhang B., Zhao K., Su C., Gong B., Ge X., Zhang Q., Li W. (2020). Comparing the multi-scale structure, physicochemical properties and digestibility of wheat A- and B-starch with repeated versus continuous heat-moisture treatment. Int. J. Biol. Macromol..

[8] Abhilasha A., Kaur L., Monro J., Hardacre A. (2022). Effects of hydrothermal treatment and low-temperature storage of whole wheat grains on in vitro starch hydrolysis and flour properties. Food Chem..

[9] Mann J., Schiedt B., Baumann A., Conde-petit B., Vilgis T.A. (2013). Effect of heat treatment on wheat dough rheology and wheat protein solubility. Food Sci. Technol. Int..

[10] Liao L., Liu H., Gan Z., Wu W. (2019). Structural properties of sweet potato starch and its vermicelli quality as affected by heat-moisture treatment. Int. J. Food Prop..

[11] Ma Y., Xu D., Sang S., Jin Y., Xu X., Cui B. (2021). Effect of superheated steam treatment on the structural and digestible properties of wheat flour. Food Hydrocoll..

[12] Villanueva M., Harasym J., María J., Ronda F. (2019). Rice flour physically modified by microwave radiation improves viscoelastic behavior of doughs and its bread-making performance. Food Hydrocoll..

[13] Shi M., Wang F., Ji X., Yan Y., Liu Y. (2022). Effects of plasma-activated water and heat moisture treatment on the properties of wheat flour and dough. Int. J. Food Sci. Technol..

[14] Gao X., Tong J., Guo L., Yu L., Li S., Yang B., Wang L., Liu Y., Li F., Guo J. (2020). Influence of gluten and starch granules interactions on dough mixing properties in wheat (*Triticum aestivum* L.). Food Hydrocoll..

[15] Wang Q., Li L., Zheng X. (2021). Recent advances in heat-moisture modified cereal starch: Structure, functionality and its applications in starchy food systems. Food Chem..

[16] Li Y., Chen W., Li H., Dong J., Shen R. (2022). Effects of Heat-Moisture Treatment Whole Tartary Buckwheat Flour on Processing Characteristics, Organoleptic Quality, and Flavor of Noodles. Foods.

[17] Chung H., Cho A., Lim S. (2014). Utilization of germinated and heat-moisture treated brown rices in sugar-snap cookies. LWT—Food Sci. Technol..

[18] Huang J., Qi Y., Manzoor M.F., Guo Q., Xu B. (2022). Effect of superheated steam treated wheat flour on quality characteristics and storage stability of fresh noodles. Food Control.

[19] Iuga M., Mironeasa S. (2020). A review of the hydrothermal treatments impact on starch based systems properties. Crit. Rev. Food Sci. Nutr..

[20] Vadlamani K.R., Seib P.A. (1996). Reduced browning in raw oriental noodles by heat and moisture treatment of wheat. Cereal Chem..

[21] Kaur A., Shevkani K., Katyal M., Singh N. (2016). Physicochemical and rheological properties of starch and flour from different durum wheat varieties and their relationships with noodle quality. J. Food Sci. Technol..

[22] Bruneel C., Pareyt B., Brijs K., Delcour J.A. (2010). The impact of the protein network on the pasting and cooking properties of dry pasta products. Food Chem..

[23] Van Hung P., Duyen T.T.M., Van Thanh H., Widiastuti D., An N.T.H. (2021). Starch digestibility and quality of cookies made from acid and heat-moisture treated sweet potato starch and wheat flour composites. J. Food Meas. Charact..

[24] Shen S., Chi C., Zhang Y., Li L., Chen L., Li X. (2021). New insights into how starch structure synergistically affects the starch digestibility, texture, and flavor quality of rice noodles. Int. J. Biol. Macromol..

[25] Evlice A.K. (2022). The effect of durum wheat genotypes on cooking quality of pasta. Eur. Food Res. Technol..

[26] Kolaric L., Minarovic L., Lauková M., Karovic J. (2020). Pasta noodles enriched with sweet potato starch: Impact on quality parameters and resistant starch content. J. Texture Stud..

[27] Biernacka B., Dziki D., Różyło R., Wójcik M., Miś A., Romankiewicz D. (2018). Relationship between the properties of raw and cooked spaghetti—New indices for pasta quality evaluation. Int. Agrophysics.

[28] Iuga M., Mironeasa S. (2021). Application of heat moisture treatment in wheat pasta production. Food Control.

[29] Ungureanu-Iuga M. (2022). Research and Contributions on the Impact of Hydrothermal Treatment of Wheat Flour and the Addition of Grape Skins on Pasta Quality.

[30] Bergman C., Gualberto D., Weber C. (1994). Development of a high-temperature-dried soft wheat pasta supplemented with cowpea (Vigna unguiculata (L) Walp)—Cooking quality, color, and sensory evaluation. Cereal Chem..

[31] Iuga M., Mironeasa S. (2021). Use of Grape Peels By-Product for Wheat Pasta Manufacturing. Plants.

[32] Giménez M.A., González R.J., Wagner J., Torres R., Lobo M.O., Samman N.C. (2013). Effect of extrusion conditions on physicochemical and sensorial properties of corn-broad beans (Vicia faba) spaghetti type pasta. Food Chem..

[33] Ungureanu-Iuga M., Dimian M., Mironeasa S. (2020). Development and quality evaluation of gluten-free pasta with grape peels and whey powders. LWT-Food Sci. Technol..

